# Glycoside hydrolase family 32 is present in *Bacillus subtilis* phages

**DOI:** 10.1186/s12985-015-0373-6

**Published:** 2015-10-06

**Authors:** Halim Maaroufi, Roger C. Levesque

**Affiliations:** Institut de biologie intégrative et des systèmes (IBIS), Plate-Forme de Bio-Informatique, Université Laval, Pavillon Charles-Eugène Marchand, 1030 Avenue de la médecine, Québec, Québec G1V 0A6 Canada; Institut de Biologie Intégrative et des Systèmes (IBIS) and Département de Microbiologie-Infectiologie et Immunologie, Faculté de Médecine, Université Laval, Québec, Québec G1V 0A6 Canada

## Abstract

**Background:**

Glycoside hydrolase family 32 (GH32) enzymes cleave the glycosidic bond between two monosaccharides or between a carbohydrate and an aglycone moiety. GH32 enzymes have been studied in prokaryotes and in eukaryotes but not in viruses.

**Findings:**

This is the first analysis of GH32 enzymes in *Bacillus subtilis* phage SP10, ϕNIT1 and SPG24. Phylogenetic analysis, molecular docking and secretability predictions suggest that phage GH32 enzymes function as levan (fructose homopolysaccharide) fructotransferase.

**Conclusions:**

We showed that viruses also contain GH32 enzymes and that our analyses *in silico* strongly suggest that these enzymes function as levan fructotransferase.

**Electronic supplementary material:**

The online version of this article (doi:10.1186/s12985-015-0373-6) contains supplementary material, which is available to authorized users.

## Findings

Bacteriophages are the most abundant organisms on Earth; estimates are at 10^31^ phage particles in the biosphere [1]. Moreover, they play a major role in the lateral gene transfer (LGT) [2]. For example, the genomes of some phages (cyanophages) have been shown to contain host-like genes known as auxiliary metabolic genes (AMGs) [3]. These genes are presumed to have been acquired from the virus’ host by lateral gene transfer (LGT) and may confer a selective advantage for persistence under certain environmental conditions [4]. The present report focuses on an AMG, a glycoside hydrolase 32 (GH32) family protein, observed for the first time in a viral genome and shown to have been acquired by LGT from the bacterial host of a phage.

Glycoside hydrolases (GH) 32 cleave the glycosidic bond between two monosaccharides or between a carbohydrate and an aglycone moiety [5]. Structurally, in addition to the catalytic five bladed β-propeller fold, GH32 enzymes are characterized by an additional β-sandwich in the C-terminal region [6]. The active site is composed of a WMNDPNG motif as the nucleophile and the EC motif as the acid/base catalyst [7]. The aspartate in the RDP motif may not to be directly implicated in the catalytic mechanism and presumably plays a role in substrate recognition and stabilization of the transition-state [8, 9].

Levan is a β-2,6-linked polymeric fructose that constitutes a carbohydrate reservoir in some plants, bacteria and fungi [10]. In microbes, it participates in the formation of the non-charged extracellular polysaccharide (EPS) matrix and plays a role in microbial biofilm formation [11]. Levan fructotransferase (LFTase; EC 4.2.2.16) is a member of GH32 family of enzymes. LFTase converts β-2,6-linked levan into DFA-IV (di-β-D-fructofuranose-2,6′:6,2′-dianhydride) [12]. DFAs production is of great importance because of their beneficial effects on human health [13]. When *Bacillus subtilis* is grown in batch cultures in sucrose-rich growth medium, it synthesizes levan [14]. In addition, Dogsa et al. [15] showed that levan, despite the fact that it is not essential for biofilm formation, could play a structural and possibly stabilizing component of *B. subtilis* floating biofilms. *Bacillus* species are found in terrestrial environments and are important industrial microorganisms used in traditional fermented foods [16, 17].

**Fig. 1:**
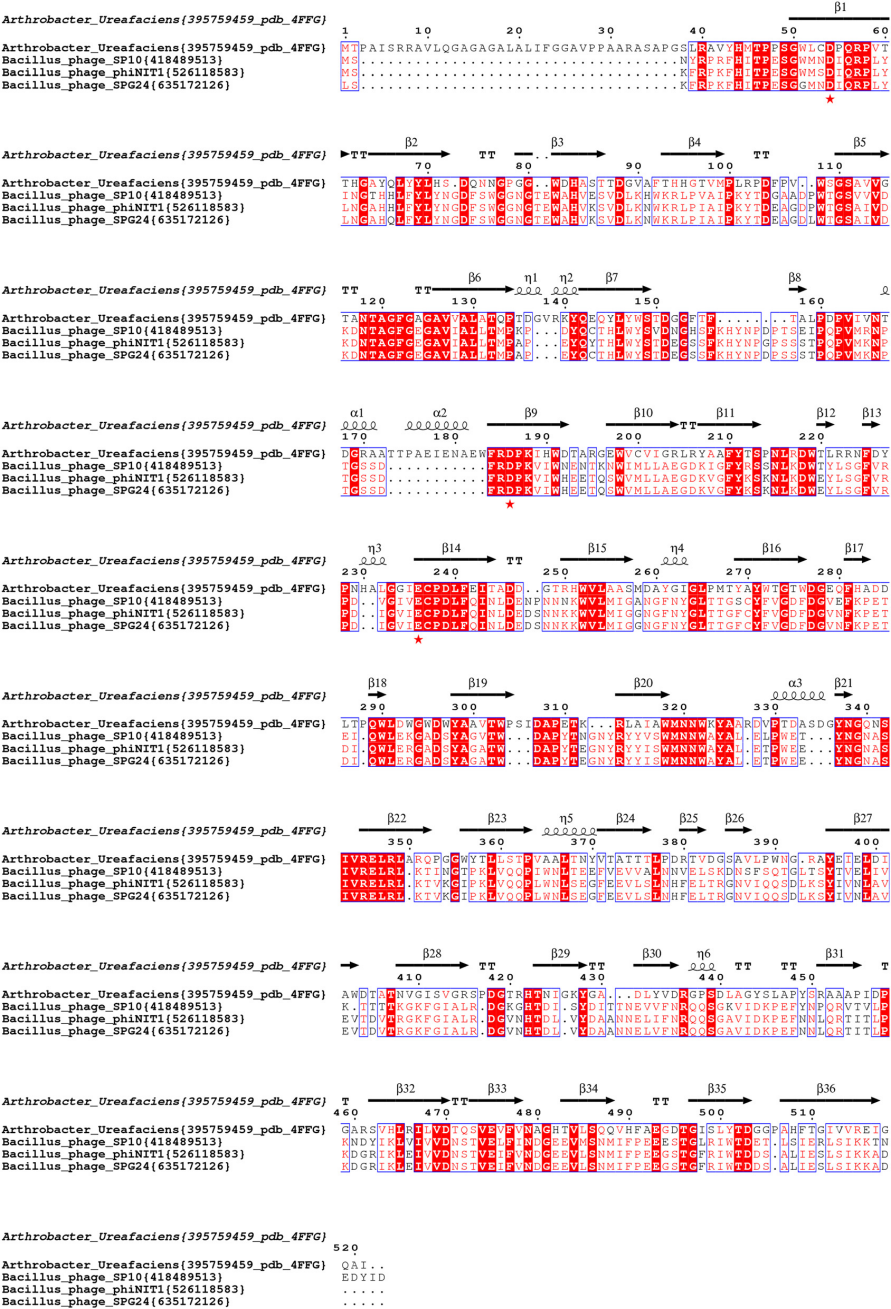
Sequence alignment of enzymes of the GH32 family from *Bacillus* phages. Accession numbers of sequence are in {} after species name. Highly conserved amino acid residues are shown in red and boxed in blue. Red asterisks represent residues of the proposed catalytic triad. Secondary structures indicated above are assigned according to the crystal structure of *A. ureafaciens* (pdb: 4FFG). The figure was prepared with ESPript (http://espript.ibcp.fr)

During our study of β-fructofuranosidase proteins of budworm (unpublished results), we noticed during the blast search that some *bacillus* phages also contain homolog to β-fructofuranosidase. The search in the CAZy database (http://www.cazy.org/) showed the presence of one *Bacillus* phage phiNIT1 sequence (accession AP013029.1) in the section of GH32 family. To determine if other homolog sequences exist in other viruses, we searched for the presence of GH32 enzyme homologs in the complete sequenced genomes of viruses in GenBank using BLASTp, tBLASTn and HMM profiles. Among 4602 (1449 phages) complete viral genomes available at NCBI (as of June 5^th^, 2015); only three *Bacillus* phage (SP10, ϕNIT1 and SPG24) genomes contain GH32 homologs. The amino acid sequence of SPG24 and ϕNIT1 shows 98 % of identity and SP10 presents 76 % of identity with SPG24 and ϕNIT1. We used the amino acid sequence of *Bacillus* phage SP10 GH32 to search by BlastP for close homologues in bacteria, fungi, plants and animals. The sequences extracted from databases were aligned with Mafft [18] (Fig. 1 and Additional file 1: Figure S1). Analysis of sequences alignment showed that in contrast to other enzymes of the GH32 family, the three enzymes from phages did not possess a signal peptide. However, using Secretome 2.0 Server (http://www.cbs.dtu.dk/services/SecretomeP/) that predicts non-classically secreted proteins in Gram-positive and Gram-negative bacteria, we obtained high scores of secretion, 0.90, 0.86 and 0.84 for enzymes from phages SP10, ϕNIT1 and SPG24 and is in accordance with secreted enzymes. Moreover, the GH32 enzymes from the three phages possess the catalytic triad, D19, D150 and E198 (Figs. 1 and 3a). We noted that in these three phage enzymes, the last three amino acid residues in the catalytic nucleophile WMNDPNG motifs are changed to IQR residues (Fig. 1 and Additional file 1: Figure S1).

**Fig. 2:**
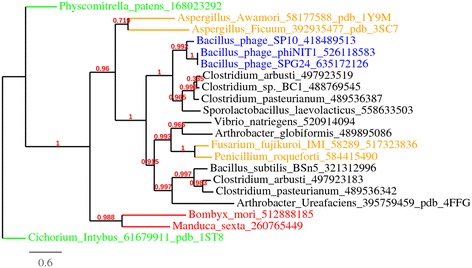
Phylogenetic relationships between the GH32 enzymes from *Bacillus* phages, bacteria, fungi, plants and animals. Sequence alignment of Additional file 1: Figure S1 was used to construct a maximum likelihood phylogenetic tree, rooted with *Cichorium* GH32. The analysis used a WAG substitution model, and the statistical confidence of the nodes was calculated using the aLRT test. GH32 enzymes from phages, bacteria, fungi, plants and animals are in blue, black, orange, green and red, respectively

To establish the phylogenetic relationships between GH32 of phages and those of prokaryotes and eukaryotes, the sequences were aligned with Mafft (Additional file 1: Figure S1) and a phylogenetic tree was constructed using PhyML [19] and BioNJ [20]. The three phage GH32 enzymes are phylogenetically closer to enzyme GH32 of *Sporolactobacillus laevolacticus* than *B. subtilis* (Fig. 2). Interestingly, *S. laevolacticus* was first isolated and described as *Bacillus laevolacticus* by Nakayama and Yanoshi [21] and confirmed by Andersch et al. [22]. In 2006, Hatayama et al. [23] phylogenetically reclassified *Bacillus laevolacticus* as *Sporolactobacillus laevolacticus* supported by chemotaxonomic and physiological characterizations. In addition to phylogeny, among 67 complete *Bacillus* phage genomes, only three contain the GH32 enzymes. Therefore, we can speculate that the *Bacillus* phages SP10, ϕNIT1 and SPG24 acquired the GH32 gene by lateral gene transfer (LGT) from *S. laevolacticus*. In metazoans, enzymes of the GH32 family are present only in arthropoda that acquired this gene from bacteria by LGT [24]. *Bacillus* phage GH32 enzymes are also close to the *Clostridium* genus. The cluster (*Clostridium* and *Sporolactobacillus*) closest to GH32 phage enzymes (Fig. 2) has the canonical catalytic nucleophile WMXDI/VQR-motif instead of the classic WMNDPNG motif (Additional file 1: Figure S1).

**Fig. 3:**
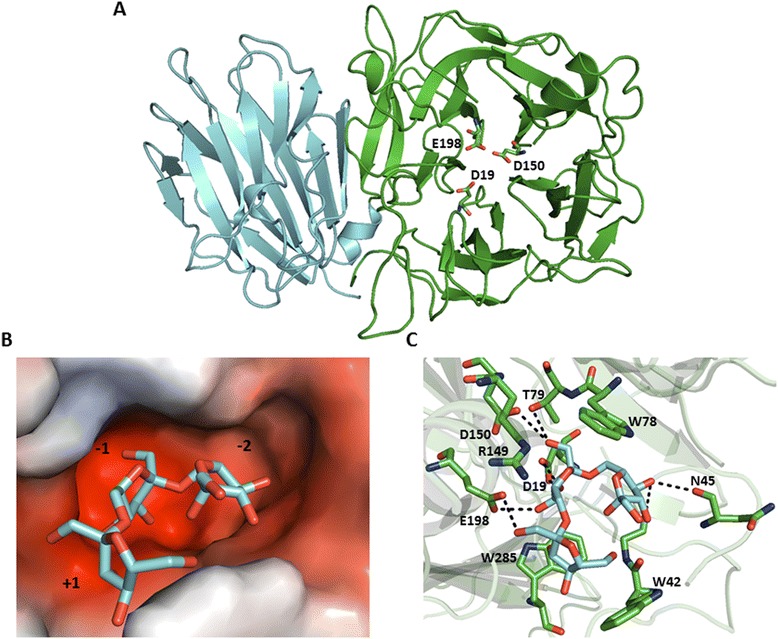
3D model of the enzyme GH32 from *Bacillus* phage SP10 and molecular docking of levantriose into the active site. **a** Cartoon view of the N- and C-domains of the GH32 enzyme from *Bacillus* phage SP10 are represented in green and cyan, respectively. Residues of the catalytic triad (D19, D150 and E198) are shown in stick representation. **b** Electrostatic potential surface representation of the active site with docked levantriose. The −1, −2 and +1 subsites are occupied by the reducing, nonreducing andfructose 3 moities, respectively. Negative and positive charges are shown in red and blue, respectively. **c** The interactions between amino acid residues in the active site and the levantriose molecule (in cyan). Hydrogen bonds are shown as black dotted lines. Images were generated using PyMol (www.pymol.org)

To begin assessing the function of the GH32 phage proteins, a three-dimensional structure model was built and the carbohydrate substrate was docked into the active site. The 3D model of phage SP10 GH32 was constructed by homology using GH32 of *Arthrobacter ureafaciens* (PDB accession: 4FFG) as template. The modeled structure of the *Bacillus* phage SP10 GH32 enzyme is similar to structures of GH32 family. The enzyme structure consists of an N-terminal domain, a five bladed β-propeller, and C-terminal, β-sandwich (Fig. 3a). The active site model contains a pocket with two compartments (subsite −1 and −2) that can accommodate a disaccharide (Fig. 3b). The active site is suitable for the exo-type cleavage of disaccharide from polysaccharide [12]. For docking, the coordinates of a levantriose carbohydrate molecule were extracted from a *A. ureafaciens* GH32 structure (PDB: 4FFI) and docked into the 3D enzyme model from phage SP10 using the software AutoDock Vina [25]. Results of the docking simulations are in accordance with the catalytic mechanism proposed for levan fructotransferase [12]. In summary, this mechanism consists of the levan substrate binding by its nonreducing terminal fructose in the −2 subsite, and the preceding fructosyl moiety acts at −1 subsite closes at the D19 nucleophile. The β-2,6-glycosidic bond between −1 and +1 subsite (interacts with the terminal fructose moiety of levantriose) and is cleaved by a nucleophilic attack by D19 followed by the E198 acid /base catalyst (Fig. 3b and c).

## Conclusion

The present study constitutes the first report of a viral GH32 enzyme, here found to be encoded by *B. subtilis* phages.

Phylogenetic analysis and molecular docking simulations strongly suggest that phage GH32 genes were acquired by LTG from an ancestral *Sporolactobacillus* host and that they function as levan fructotransferase. These observations suggest that phage GH32 enzymes could be used therapeutically to destroy microbial biofilms. Indeed, it has been shown *in vitro* that phages are able to infect biofilm cells and induce the production of depolymerases that degrade components of the biofilm exopolymeric matrix [26, 27]. Phage GH32 proteins could also be used in industry to produce DFAs that have beneficial effects on human health [13].

## Additional file

Additional file 1: Figure S1. Sequence alignment of enzymes of the GH32 family from *Bacillus* phages and close homologs of the GH32 enzyme family. The amino acid sequences were compared to close homologs of the GH32 enzyme family found in bacteria, fungi, plants and animals. Accession numbers of sequence are in {} after species name. The GH32 enzyme structures from *Arthrobacter ureafaciens* (pdb: 4FFG), *Aspergilus awamori* (pdb: 1Y9M) and *Aspergilus ficuum* (pdb: 3SC7) were used in the alignment to help choose the most appropriate template for homology 3D model construction (see Fig. 3). Highly conserved amino acid residues are shown in red and boxed in blue. Red asterisks represent residues of the proposed catalytic triad. Secondary structures indicated above are assigned according to the crystal structure of *A. ureafaciens* (pdb: 4FFG) resolved at 2.30 Å. The figure was prepared with ESPript (http://espript.ibcp.fr). (PDF 21 kb)

## Abbreviations

GH32: Glycoside hydrolase family 32
AMGs: Auxiliary metabolic genes
LGT: Lateral gene transfer
EPS: Extracellular polysaccharide
LFTase: Levan fructotransferase
DFA-IV: di-β-D-fructofuranose-2,6′:6,2′-dianhydride
