# Supplementary material for: Glycoside hydrolase family 32 is present in Bacillus subtilis phages
Source: Virol J. 2015 Oct 6;12:157. doi: 10.1186/s12985-015-0373-6 (PMC4595243; doi:10.1186/s12985-015-0373-6)
Supplement: Additional file 1: Figure S1. — Sequence alignment of enzymes of the GH32 family from Bacillus phages and close homologs of the GH32 enzyme family. The amino acid sequences were compared to close homologs of the GH32 enzyme family found in bacteria, fungi, plants and animals. Accession numbers of sequence are in {} after species name. The GH32 enzyme structures from Arthrobacter ureafaciens (pdb: 4FFG), Aspergilus awamori (pdb: 1Y9M) and Aspergilus ficuum (pdb: 3SC7) were used in the alignment to help choose the most appropriate template for homology 3D model construction (see Fig. 3). Highly conserved amino acid residues are shown in red and boxed in blue. Red asterisks represent residues of the proposed catalytic triad. Secondary structures indicated above are assigned according to the crystal structure of A. ureafaciens (pdb: 4FFG) resolved at 2.30 Å. The figure was prepared with ESPript (http://espript.ibcp.fr). (PDF 21 kb) [file 12985_2015_373_MOESM1_ESM.pdf]

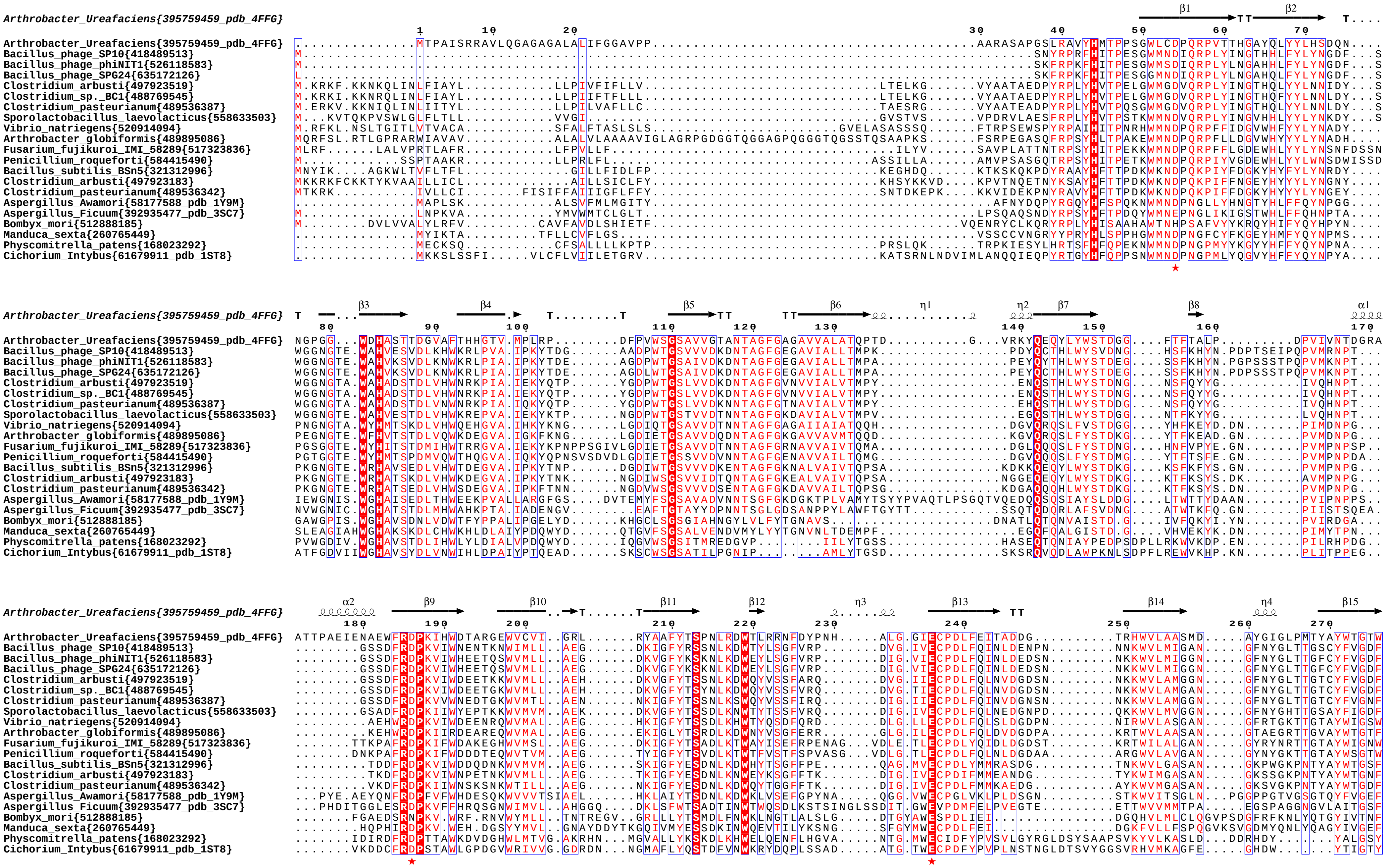
Arthrobacter_Ureafaciens{395759459_pdb_4FFG}
Bacillus_phage_SP10{418489513}
Bacillus_phage_phiNIT1{526118583}
Bacillus_phage_SPG24{635172126}
Clostridium_arbusti{497923519}
Clostridium_sp._BC1{488769545}
Clostridium_pasteurianum{489536387}
Sporolactobacillus_laevolacticus{558633503}
Vibrio_natriegens{520914094}
Arthrobacter_globiformis{489895086}
Fusarium_fujikuroi_IMI_58289{517323836}
Penicillium_roqueforti{584415490}
Bacillus_subtilis_BSn5{321312996}
Clostridium_arbusti{497923183}
Clostridium_pasteurianum{489536342}
Aspergillus_Awamori{58177588_pdb_1Y9M}
Aspergillus_Ficuum{392935477_pdb_3SC7}
Bombyx_mori{512888185}
Manduca_sexta{260765449}
Physcomitrella_patens{168023292}
Cichorium_Intybus{61679911_pdb_1ST8}
β1 β2 β3 β4 β5 β6 η1 η2 β7 β8 α1 α2 β9 β10 β11 β12 η3 β13 β14 η4 β15

```
                                             β16        β17        β18        β19        α3   β20      β21        β22       η5   β23        β24        β25
Arthrobacter_Ureafaciens{395759459_pdb_4FFG}
                                          280          290         300         310         320         330         340         350         360         370         380

Arthrobacter_Ureafaciens{395759459_pdb_4FFG}  ..DGEQFHADD.......LTPQWLDWGWDWYAAVTWPSIDAPET....KRLAIAWMNNWKYAARDVPTDASDGYNGQNSIVRELRLARQPGGWYTLLSTPVAALTNYVTATTTLPD..RTV.D..GSAVL...P
Bacillus_phage_SP10{418489513}                ..DGVEFKPET........EIQWLEKGADSYAGVTWDAPYTNGN....YRYYVSWMNNWAYAL.ELPW..ET.YNGNASIVRELRLKTIN.GTPKLVQQPIWNLTEEFVEVVALNN..VEL....SKDNS.FSQ
Bacillus_phage_phiNIT1{526118583}             ..DGVNFKPET........DIQWLERGADSYAGATWDAPYTEGN....YRYYISWMNNWAYAL.ETPW..EE.YNGNASIVRELRLKTVK.GIPKLVQQPLWNLSEGFEEVLSLNH..FEL....TRGNV.IQQ
Bacillus_phage_SPG24{635172126}               ..DGVNFKPET........DIQWLERGADSYAGATWDAPYTEGN....YRYYISWMNNWAYAL.ETPW..EE.YNGNASIVRELRLKTVK.GIPKLVQQPLWNLSEGFEEVLSLNH..FEL....TRGNV.IQQ
Clostridium_arbusti{497923519}                ..DGQTFTQDT......GSDISWLEEGADSYAGVTWDTPYTNGN....YRYFISWMNNWNYAL.ELPW..EN.YSGNASVVRELRLKTTS.NGLKLQEVPVWNLLDNFTEVINIEN..ETVYT..NQENI.LKN
Clostridium_sp._BC1{488769545}                ..DGQTFTPDT......GSDISWLEEGADSYAGVTWDTPYTNGN....YRYFISWMNNWNYAL.QIPW..NN.YSGNASIVRELRLNTTI.NGLKLQEVPVWNLLSNFNQVMDIEN..QTIYT..NQSNI.LDS
Clostridium_pasteurianum{489536387}           ..DGEVFTKDP......GSDLNWLEEGADSYTGVTWDAPYTDGN....YRYFISWMNNWNYAF.QLPW..QN.YSGNTSIVREIRLNTTS.NGLKLQQVPVWNLLSNFNEVMNVEN..QTVYN..NQQNI.LDN
Sporolactobacillus_laevolacticus{558633503}   ..DGESFHNQT........NVKWLEDGPDSYTGVTWDAPYTNEN....YRYFISWMSNWNYAR.QVPW..EN.YIGNTSVVRELTLRNTS.EGLKLVQNPVWNLKDNLTELAYFGD..QVIYT..GQDNL.LKN
Vibrio_natriegens{520914094}                  ..DGKRFIPES.......EEPQWLDAGADFYAMVSWQDSNLGADQRLESRYAIGWLNNWGYAN.ELPT..KAWHGAASSIVRQIKLRTVD.GTPVLFSQPIEAIAGLEGDAYTRSA..VEV.LESSNTSF..PK
Arthrobacter_globiformis{489895086}           ..DGTTFTTAD.......DKHQWLDAGADFYAAVTWDDPRLTENERMASRHAIGWMNNWTYAR.QLPT..TDWQGGADSIVRDIRLKTVQ.GRPTLVSTPTPALSALDGEAKTADS..GAL.TPEGAGGL..PA
Fusarium_fujikuroi_IMI_58289{517323836}       ..DGNRFTATE.......SFPQWMDDGPDFYAAVSWENP....TDRYGSRYAIAWMNNWDYAA.SLPY..YADFAGQDSLVREVELKTIN.GSPTLVSIPIGGYEEILASPMSVSD..KTITTDPASASL.PSG
Penicillium_roqueforti{584415490}             ..DGTNFTPTT.......SVPQWLDEGPDFYATVSWENP....DDKFGSRYAIGWMNSWNYAS.YLPY..YGDFEGQQSLAREIKYQTIN.GTPMLVSLPVSGYADLFAGPVSADG..TEITTDPATASL.PAD
Bacillus_subtilis_BSn5{321312996}             ..DGKEFKADQ.......TEAQWLDYGFDWYGGVTFEDSKSTDP..LEKRYALAWMNNWDYAN.NTPT.MKNGFNGTDSVIRELRLKEQD.GTYSLVSQPIEALEQLTVSTDEIED..QDV.N..GSKTL...S
Clostridium_arbusti{497923183}                ..NGKEFVPDE.......IEPQWLDYGFDWYAGVTFDSGNSSDK..YTKRYALAWMNNWDYPN.NTPT.LKDGFNGMDSIVRQISLKKQDNDTYSLISQPVENLSGFTKSTEHLKK..IEV.T..GSKTL...K
Clostridium_pasteurianum{489536342}           ..NGKEFIPDE.......SEPQWLDYGFDWYAGVTFESGNISES..FIKRYALAWMNNWDYAN.NTPT.TKENFNGIDSIVREISLKKQD.DRYSLFSQPVETLNDIVKSTEHLNR..VEV.T..GTKTL...E
Aspergillus_Awamori{58177588_pdb_1Y9M}        ..DGTTFTPDADTVYPGNSTANWMDWGPDFYAAAGYNGLS......LNDHVHIGWMNNWQYGA.NIPT..YP.WRSAMAIPRHMALKTIG.SKATLVQQPQEAWSSISNKRPIYSRTFKTL.SE.GSTNT...T
Aspergillus_Ficuum{392935477_pdb_3SC7}        ..DGKSFTADP.....VDASTMWLDNGRDFDGALSWVNVPASD....GRRIIAAVMNS..YGS.NPPT..TT.WKGMLSFPRTLSLKKVG.TQQHFVQQPITELDTISTSLQILAN..QTI.T..PGQTL.LSS
Bombyx_mori{512888185}                        NYVNGQFDDLE....VSTATFTELDHGHDFYAPKTVLAVD.......GRRLLIGWLGMWESHF.KESK..HG.WASMLTIVREMKLTPQG....RLLMPPVREVAELRTEILEDAWY.....N..PGEAF....
Manduca_sexta{260765449}                      DYDTHSFTILT.......EFRELDHGHDFYATQTMKDPS.......GRRIVVAWASTWEYAY.PERA..DG.WAGMLTLPRTLTLT....KDLRLIQTPIREIDQVFRRRLYSGKA.....S..AGKTV...A
Physcomitrella_patens{168023292}              NVKSKSFHADDPSRDTG..IGLRYDYG.KFYASKSFYDAA......QQRRILWGWANESDSEA.ADYA..KG.WSSVQAIPRTIRYDSK..TMRNLIQEPVEELKELRGPRVSQKS..VRL.A..PGSVVEVHG
Cichorium_Intybus{61679911_pdb_1ST8}          SPDRENFLPQNGLSLTGSTLDLRYDYG.QFYASKSFFDDA......KNRRVLWAWVPETDSQA.DDIE..KG.WAGLQSFPRALWIDR...NGKQLIQWPVEEIEELRQNQVNLQN..KNL.K..PGSVLEIHG

                                                 β26                 β27                      β28      β29    η6               β30        β31     β32
Arthrobacter_Ureafaciens{395759459_pdb_4FFG}                                                  TT            TT              TT    T........T     TT          TT
                                          390       400                          410         420         430         440         450         460         470

Arthrobacter_Ureafaciens{395759459_pdb_4FFG}  WNGRAYEIELDIAWDTATNV...........................GISVGRSPDG..TRHTNIGK..YGAD.......LYVDRGPSDLAGYSLA........PYSRAAAPIDPGA.RSVHLRILVDTQSVEV
Bacillus_phage_SP10{418489513}                TGLTSYTVELIVKTT.TTKG...........................KFGIALRDGK...GHTDISYDITTNE.......VVFNRQQSGK.VIDKP.......EFYNPQRVTVLPKN.DYIKLVIVVDNSTVEL
Bacillus_phage_phiNIT1{526118583}             SDLKSYIVNLAVEVTDVTRG...........................KFGIALRDGV...NHTDLVYDAANNE.......LIFNRQQSGA.VIDKP.......EFNNLQRTITLPKD.GRIKLEIVVDNSTVEI
Bacillus_phage_SPG24{635172126}               SDLKSYIVNLAVEVTDVTRG...........................KFGIALRDGV...NHTDLVYDAANNE.......LVFNRQQSGA.VIDKP.......EFNNLQRTITLPKD.GRIKLEIVVDNSTVEI
Clostridium_arbusti{497923519}                YKGLSYSIETEIDVSDLTNG...........................KFGFSIRSGL..DEHTDITYDKSLNE.......LSFDRSNSGI.IVNVE.......EFTRPQKVTVNPVN.GKIKLTILVDASTVEI
Clostridium_sp._BC1{488769545}                YKGLSYSIETQIDVSDLTNG...........................KFGFSVRCGL..NEQTNITYDKSINQ.......LSFDRSNSGT.IVNVN.......EFTSPQRVTADPIN.GKIKLTILVDVSTVEI
Clostridium_pasteurianum{489536387}           YKGLSYSIETQIDVSDLTNG...........................KFGFSVRNGL..NEHTDITYDKSINQ.......LSFDRSNSGI.VVNVN.......ESINLQRITVNPVN.GKIKLTILVDVSTVEI
Sporolactobacillus_laevolacticus{558633503}   IHETSYSIESTFTVDDSTSA...........................KFGLSLRDGN..DEHTDITYDKNIQE.......LVFDRTHSGI..VSMT.......DFAKAYKVHIPPKN.GKIKLDILVDRSTIEI
Vibrio_natriegens{520914094}                  PISDAYRLKVDLDAH..SNA..........................SEFQLQLKGKD..GHFAIVGYDFEHET.......VFIRRDRDAI.ASSMP.......DVYRDERKTVVRAHNGIVKLDIIVDTFTIEV
Arthrobacter_globiformis{489895086}           PAGGAYRLDVTLERAPGDDG..........................TEALLKLGNGG..ADFATVGYNFEAGT.......AWVDRAAVVPGADALGPVFTERRSAQSPPRNGSSPGR.GSVELTIFVDYSSAEV
Fusarium_fujikuroi_IMI_58289{517323836}       MEQGAYMIRATISKNGGDKG..........................NEVRFVIKSDG..AFSTTVGYDFVHSQ.......AFLVRDSDGSATDSMAA...GSKQVYDTVRTAPDPSGGSTVKLTIYVDWNSVEI
Penicillium_roqueforti{584415490}             LAGGAYVIQATVSKADGDDG..........................NTVYFRIKSDG..TYNTTVGYDFANSQ.......LFLVRDSDGSASDVLTS...DAKQTWDVTRTATNPAGGDTVQLTIYVDWNSVET
Bacillus_subtilis_BSn5{321312996}             ITGDTYQLDTDLSWSELKNA...........................GVRLRESEDQ..KRHIDVGIFAEGGY.......AYVNRAATNQ..PDKS.......NTYVESKAPYDVNK.RKVHLKILVDKTTIEV
Clostridium_arbusti{497923183}                VKGDAYEIDADISWSDAKNV...........................GFRLRESEDK..NRHIDVGVFVEGQY.......SYVNRAFTGQ..PDKT.......EKYVESKAHFDTSK.KKVHLKILVDKTSVEV
Clostridium_pasteurianum{489536342}           FTGESYELDADISWEDAENV...........................GLRLRESEDK..SRHIDVGIFVDGKY.......SYVNRGFTSH..PDAA.......NKYIESRAPFDTSK.KKVHLKIIVDKTSIEV
Aspergillus_Awamori{58177588_pdb_1Y9M}        TTGETFKVDLSFSAKS.KAS...........................TFAIALRASANFTEQTLVGYDFAKQQ.......IFLDRTHSGDVSFDET........FASVYHGPLTPDSTGVVKLSIFVDRSSVEV
Aspergillus_Ficuum{392935477_pdb_3SC7}        IRGTALDVRVAFYPDAG..S...........................VLSLAVRKGA..SEQTVIKYTQSDAT.......LSVDRTESGDISYDPA........AGGVHTAKLEEDGTGLVSIRVLVDTCSVEV
Bombyx_mori{512888185}                        .YAGTRTFELIVSTAK............................VFYDVAVIFEWDG..DQQYTVGYSADRGH.......IIVDRGGVDG.................LRRADWAPN..DKLKLRIFVDYSSIEV
Manduca_sexta{260765449}                      LPDKAGKVELKWDTPR...............................NIKVVIESQNE.CQNVVISYDHEDGT.......ITLDRGGDDA.................IRRTHWDPR..GHLKWTIFIDASSIEL
Physcomitrella_patens{168023292}              AIGGQLDIEVVIEYPNVTKLSQNGALIDDGDHFDCSQGGAAHRGTFGPFGLLVLADESLNERTAVFFYISYSKEGKWRTRLCSDQTKSSM.LPDVD.......TTIYGSFVEVLPSE.DFLSLRVLVDRSIVES
Cichorium_Intybus{61679911_pdb_1ST8}          IAASQADVTISFKLEGLKEAEVLDTTLVDPQAL.CNERGASSRGALGPFGLLAMASKDLKEQSAIFFRVFQNQLGRYSVLMCSDLSRSTV.RSNID.......TTSYGAFVDIDPRS.EEISLRNLIDHSIIES

                                                   β33              β34        β35
Arthrobacter_Ureafaciens{395759459_pdb_4FFG}                       TT
                                              480       490       500       510       520

Arthrobacter_Ureafaciens{395759459_pdb_4FFG}  FVNAGHTVLSQQVHFA...EGDTGISLYTDGG.PAHFTGIVVREIGQAI.................
Bacillus_phage_SP10{418489513}                FINDGEEVMSNMIFPE...EESTGLRIWTDE..TLSIERLSIKKTNEDYID...............
Bacillus_phage_phiNIT1{526118583}             FVNDGEEVLSNMIFPE...EGSTGFRIWTDD..SALIESLSIKKAD....................
Bacillus_phage_SPG24{635172126}               FVNDGEEVLSNMIFPE...EGSTGFRIWTDD..SALIESLSIKKAD....................
Clostridium_arbusti{497923519}                FVNDGEHTLSNIIFPK...LISDEVRLWTDD..HVHLDYLKIRNNDINRIKAHSNK..........
Clostridium_sp._BC1{488769545}                FVNDGQYTLSNIIFPS...LTSNGVRLWTDD..HVHLDYLKIRNNDNNMIN...............
Clostridium_pasteurianum{489536387}           FVNDGQHTLSNIIFPS...LTSDGVRLWTDG..HVHLDYLKIRNNNNNMIN...............
Sporolactobacillus_laevolacticus{558633503}   FVNDGEYVLSSLIFPQ...LSSDGLRLWTDD..HVHIDYLKIRKADNSTID...............
Vibrio_natriegens{520914094}                  FVNDGEMSLSNLIFGT...QDANDLTAVSVGG.TTVLRNLELTPLKVTPIQRYSGEGAKK......
Arthrobacter_globiformis{489895086}           FVNGGEQTLTSVVLPR...PGQPAVSAATAGG.KLTLKSFKYTPLATVLTAR..............
Fusarium_fujikuroi_IMI_58289{517323836}       FVNDGVAVLSGLIYPN...QGASGVQVVSDTG.SLTLVSFSYAACGSIY.................
Penicillium_roqueforti{584415490}             FVNGGVAALSALIYPN...QDAEDIEVVSDSG.KLTLTSFSYAGFQV...................
Bacillus_subtilis_BSn5{321312996}             FVGDGKTVFSNEVFPK...PEDKGITLYSDGG.TASFKNITVKHFDSIHE................
Clostridium_arbusti{497923183}                FVDDGKVTYSNEVFPD...LNDKGISLFSVGG.KSIFENVQIKHFR....................
Clostridium_pasteurianum{489536342}           FVDDGKVTYSNEAFPD...LKDKGISLYSIGG.KAVFENIQIKHFDSINEKD..............
Aspergillus_Awamori{58177588_pdb_1Y9M}        FGGQGETTLTAQIFPS...SDAVHARLASTGG.TTEDVRADIYKIASTW.N................
Aspergillus_Ficuum{392935477_pdb_3SC7}        FGGQGEAVISDLIFPS...DSSDGLALEVTGG.NAVLQSVDVRSVS...LE...............
Bombyx_mori{512888185}                        FCGSGDVVFSSRVYPK....KNIRVKI...SG.ESQLHVVQYKLRRSVGYDSKLRKYLKEHVLERA
Manduca_sexta{260765449}                      SCGDGEVWFTSRFFPE....GVVSVRL...GE.DTCVDKFTVHSIRRTTPDPE....AHCRCESEE
Physcomitrella_patens{168023292}              FGQGGRMTITSRVYPTMATDTASHLYLFNNATTAITVRSIDVWQMRSVAMHAI.............
Cichorium_Intybus{61679911_pdb_1ST8}          FGAGGKTCITSRIYPKFVNNEEAHLFVFNNGTQNVKISEMSAWSMKNAKFVVD......QSVKSAA
```
